# The log-linear failure rate distribution: properties, estimation and application to aircraft windshield failure and service data

**DOI:** 10.1038/s41598-026-45633-8

**Published:** 2026-04-22

**Authors:** C. M. Revathi, Rajesh Moharana

**Affiliations:** Department of Mathematics, School of Advanced Sciences, Vellore Institute of Technology, Vellore, Tamil Nadu 632014 India

**Keywords:** The Log-Linear Failure Rate Distribution, Properties, Stochastic Ordering, Estimation, Real Data Application, Engineering, Mathematics and computing

## Abstract

This study proposes the Log-Linear Failure Rate (Log-LFR) distribution, a novel extension of the classical Linear Failure Rate model achieved through a logarithmic transformation. The suggested logarithmic generator is characterized by its survival-based construction, permitting a natural hazard interpretation and an effortless recovery of the baseline model as the logarithmic parameter tends to one. It improves tail flexibility and accommodates various hazard behaviors while preserving analytical flexibility and symmetry. While several extensions of the Linear Failure Rate distribution exist, the logarithmic transformation has not been explored before. This addresses a gap in the literature and provides more flexibility in modeling diverse aging patterns and failure behaviors. We investigate the statistical and reliability properties of the proposed Log-LFR distribution such as moments, moment generating function, quantile function, order statistics and reliability measures. Further, stochastic comparisons are studied under sufficient conditions to provide theoretical insights into the distribution’s behavior. Parameters are estimated using Maximum Likelihood Estimation. Their performance is validated through extensive Monte Carlo simulations showing accuracy and robustness. The model is applied to two real-world datasets on aircraft windshield failure and service times. In both instances, it shows a better fit than other competing models. These results show the Log-LFR distribution as a versatile and effective tool for reliability and survival data analysis in industrial and aerospace applications.

## Introduction

The lifetime models are popular statistical tools for fitting and analyzing survival data in many fields, especially in the field of engineering and survival sciences. There are many multi-parameter distributions in statistical theory to fit different types of data. As mentioned in the research article, in recent decades, many new families of lifetime distributions have been developed to fit complex data sets in which traditional distributions cannot adequately describe complex patterns. The Linear Failure Rate (LFR) distribution is a common distribution model in reliability engineering for characterizing components or systems exhibiting monotonically linear failure rates. It makes easier to figure out how reliable a system is and gives a more complete picture of how the system works. The probability density function (pdf), cumulative distribution function (cdf), survival function (sf) and hazard rate function (hrf) of the LFR distribution are, respectively, defined below:

1 $$\begin{aligned} f_{LFR}(x)=(\alpha +\beta x)\exp \left\{ -\left( \alpha x+\frac{\beta }{2}x^2\right) \right\} , \end{aligned}$$

2 $$\begin{aligned} F_{LFR}(x)=1-\exp \left\{ -\left( \alpha x+\frac{\beta }{2}x^2\right) \right\} , \end{aligned}$$

3 $$\begin{aligned} \bar{F}_{LFR}(x)=\exp \left\{ -\left( \alpha x+\frac{\beta }{2}x^2\right) \right\} , \end{aligned}$$

4 $$\begin{aligned} \text{ and }~~h_{LFR}(x)=\alpha + \beta x, \end{aligned}$$

where $x>0$, $\alpha>0$ is the shape parameter, and $\beta>0$ is the scale parameter. The exponential distribution and Rayleigh distribution can be obtained from the LFR distribution by taking $\beta =0$ and $\alpha =0$, respectively. Additionally, the pdf of the LFR distribution may be either unimodal or decreasing, whereas the failure rate function is exclusive to either constant or increasing. The following literature provides detailed discussions and results concerning the LFR distribution.

Kodlin^1^ was among the first to apply the LFR distribution to human lifetime data, thereby initiating its relevance in reliability analysis. Subsequently, Bain^2^ advanced the study of life-testing distributions by employing polynomial hazard functions and advocating for the use of maximum likelihood estimation as a more efficient method for parameter estimation. Bayesian inference for the LFR distribution under progressive censoring schemes was later explored by Sen and Bhattacharyya^3^. In recent years, several generalizations of the LFR distribution have been proposed to enhance its flexibility for modeling diverse types of data. For instance, Cordeiro et al.^4^ introduced the gamma-linear failure rate distribution, and Sarhan and Kundu^5^ developed the generalized linear failure rate distribution. Mahmoudi and Jafari^6^ proposed the LFR–power series distribution, while Jafari and Mahmoudi^7^ later introduced the Beta-LFR distribution, a four-parameter generalized model. Najwan Alsadat et al.^8^ contributed the truncated Cauchy power LFR distribution, and Mahdy and Telbany^9^ formulated a weighted version of the LFR distribution. Elbatal et al.^10^ extended the research by introducing the McDonald generalized LFR distribution, which is a six-parameter distribution model with substantial flexibility for use in survival analysis and reliability theory. Bui et al.^11^ introduced the LFR odd ratio-G distribution model, which is a mixture of exponentiated odd ratio and LFR distribution for modeling bathtub-shaped hazard rate. Ramkiran et al.^12^ proposed an auxiliary function approach using relative measures of dispersion for deriving closed-form expressions of the parameters of the LFR distribution. Ratnasingam and Ning^13^ introduced a change point detection method for the LFR distribution under random censorship, where asymptotic results were established and confidence sets were developed by using confidence distribution theory. While numerous extensions of the LFR distribution have been proposed using various generator-based approaches, still other types of generalizations remain possible. Our work builds on this idea by introducing a logarithmic transformation applied to the LFR distribution. This is an area that has not been addressed in any previous literature.

Although the LFR distribution is most suitable in modeling situations where the failure rate increases over time, there are situations where the failure rate is non-monotonic. Situations like this are common in biological systems and in firmware reliability modeling, as observed in the works of Lai and Xie^17^ and Zhang et al.^18^. Inspired by the logarithmic generalization technique proposed by Pappas et al.^14^, which extends the foundational methodology of Marshall and Olkin^15^, we propose a novel variant of the LFR distribution to effectively model such scenarios. This approach introduces an additional shape parameter $p>0$ into the survival function to increase distributional flexibility. Let $\bar{F}(x)$ denote the survival function of a baseline distribution. The generalized sf $\bar{G}(x)$ is then defined as

5 $$\begin{aligned} \bar{G}(x)=\frac{\ln (1-(1-p)\bar{F}(x))}{\ln (p)},~ x>0,~ p>0. \end{aligned}$$

This transformation modifies the tail behavior of the distribution. This enables the distribution to capture different rates of tail decay based on the value of *p*. It is interesting to note that, when $p\rightarrow 1$, the transformed survival function $\bar{G}(x)$ converges to the original $\bar{F}(x)$. This implies that the baseline model is retained. The transformation results in new expressions for the pdf and hrf. The new expressions for the pdf and hrf combine both the original and new parameter *p*. The expression for the pdf of the transformed model is given by

6 $$\begin{aligned} g(x)=\frac{(p-1)f(x)}{(1-(1-p)\bar{F}(x))\ln (p)}, ~x>0,~ p>0, \end{aligned}$$

and the hazard rate function becomes

7 $$\begin{aligned} h(x)=\frac{(p-1)\bar{F}(x)\gamma (x)}{(1-(1-p)\bar{F}(x))\ln (1-(1-p)\bar{F}(x))},~ x>0, ~p>0, \end{aligned}$$

where *f*(*x*) and $\gamma (x)$ denote the pdf and hrf of the baseline distribution, respectively. This generalized framework enhances the modeling capability by introducing controllable skewness and tail behavior, making it particularly suitable for lifetime and reliability data exhibiting non-standard hazard rate shapes. Motivated by recent advances in lifetime data modeling, Alzawq et al.^21^ proposed the Log-Lindley distribution as a flexible two-parameter extension of the classical Lindley distribution. This new model was developed by compounding the Lindley distribution with a logarithmic transformation, which enhanced its capability to capture various shapes of hazard rate functions observed in practical scenarios. The authors conducted a thorough investigation of its statistical properties, including moments and reliability characteristics, and utilized maximum likelihood estimation techniques for parameter inference. Their simulation studies further confirmed that the proposed estimators exhibit desirable properties such as consistency and efficiency. Inspired by this framework, we introduce a novel distribution called the Log-Linear Failure Rate (Log-LFR) distribution. This model extends the classical linear failure rate distribution by incorporating a logarithmic transformation. The transformation enhances flexibility in modeling complex failure behaviors and improves the fit for various reliability datasets. Based on this development, the primary objectives of the study are outlined as follows:

(i) To propose the Log-LFR distribution as a generalized extension of the classical LFR distribution through a logarithmic transformation.
(ii) To study the statistical and reliability properties of the proposed Log-LFR distribution.
(iii) To evaluate the effectiveness of Maximum Likelihood Estimation for parameter estimation and assess the statistical properties of the estimators through simulations.
(iv) To illustrate the practical usage of the Log-LFR distribution using real-life datasets on aircraft windshield failures and service times, and to compare its performance with alternative lifetime distributions based on standard goodness-of-fit measures.

The content of the paper is structured as follows. First, we introduce the Log-LFR distribution as a novel generalization of the classical LFR model. A comprehensive analysis of its statistical and reliability properties is then presented, including the hazard rate function, reversed hazard rate, mean residual life function, moments, moment-generating function, quantile function, distribution of order statistics, and Rènyi entropy. Thereafter, we explore some stochastic ordering results, especially on systems with components having this new Log-LFR distribution. Illustrative examples and counterexamples are provided to support the theoretical findings. Next, a Monte Carlo simulation study is carried out in order to assess the performance and estimation accuracy of the proposed model. The practical applicability of the Log-LFR distribution is then demonstrated through real-world reliability data on aircraft windshields. The paper is then concluded with a summary of all the findings as well as some possible avenues for future research.

## The log-linear failure rate distribution

This section is devoted to the construction of the Log-LFR distribution, followed by an in-depth study of the statistical properties of the newly developed distribution, including moments, the moment generating function, the quantile function, as well as some of the most important reliability properties, namely the hazard rate function and the mean residual lifetime function. The analysis highlights the flexibility of the proposed distribution in modeling diverse failure patterns. Additionally, the section explores order statistics and Rényi entropy to further support its applicability in reliability analysis.

The survival function given in equation (3) is utilized in equation (5) to extend the traditional LFR distribution by introducing a new shape parameter $p>0$. Upon simplification, this leads to the survival function of the proposed Log-LFR distribution as presented below:

8 $$\begin{aligned} \bar{F}_{Log-LFR}(x)=\frac{\ln \Big (1-(1-p)e^{-\big (\alpha x +\frac{\beta }{2} x^2\big )}\Big )}{\ln (p)}, ~ x>0,~ \alpha , \beta , p>0. \end{aligned}$$

Then, the probability density function of Log-LFR distribution corresponding to equation (8) is readily found to be

9 $$\begin{aligned} f_{Log-LFR}(x)=\frac{(p-1)\big (\alpha +\beta x\big )e^{-\big (\alpha x +\frac{\beta }{2} x^2\big )}}{\Big (1-(1-p)e^{-\big (\alpha x +\frac{\beta }{2} x^2\big )}\Big )\ln (p)}, ~x>0, ~\alpha , \beta , p>0. \end{aligned}$$

In the following, we present the plots of the pdf and cdf of the Log-LFR distribution for various parameter values to facilitate visualization and illustrate the distribution’s behavior.

**Fig. 1:**
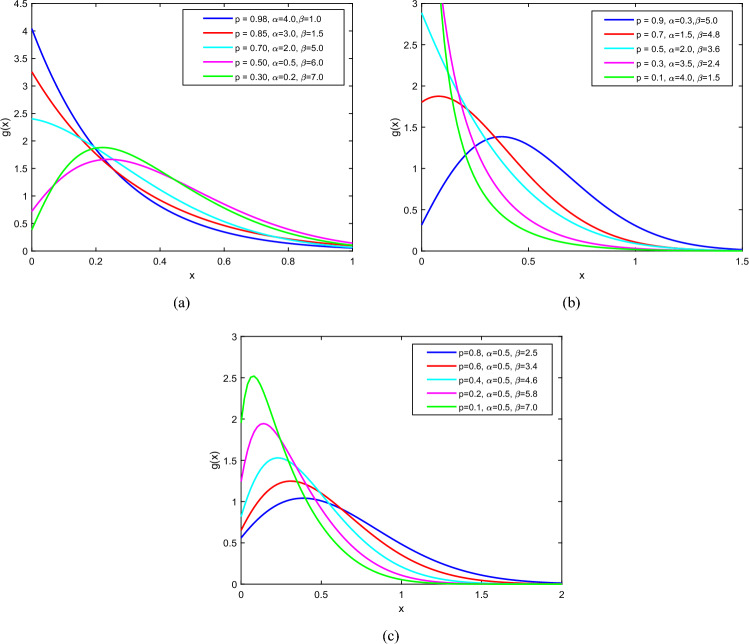
(**a**), (**b**) and (**c**) illustrate different shapes of the pdf of the Log-LFR distribution corresponding to specific parameter values.

**Fig. 2:**
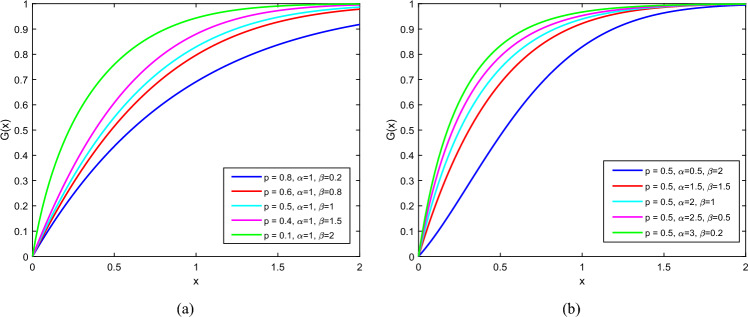
(**a**) and (**b**) represent various patterns of the cdf of the Log-LFR distribution for specified parameter values.

Figure 1 demonstrates that the Log-LFR distribution can exhibit both monotonically and positively skewed pdf shapes depending on the choice of parameter values and Fig. 2 represents the plot of the cdf of the Log-LFR distribution for different parameter values.

### Moments

In the following, we derive *r*-th moment of the Log-LFR distribution which is given by

10 $$\begin{aligned} \mu ^{'}_{r}=E(X^{r}) =\frac{(p-1)}{\ln (p)}\int _{0}^{\infty } x^{r}\left( \frac{\big (\alpha +\beta x\big ) e^{-\big (\alpha x+\frac{\beta }{2}x^2\big )}}{1-(1-p)e^{-\big (\alpha x+\frac{\beta }{2}x^2\big )}}\right) dx. \end{aligned}$$

Now, set $z=\alpha x+\frac{\beta }{2}x^2$, which implies that $x=\frac{1}{\beta }\Big (\big (\alpha ^2+2\beta z\big )^{\frac{1}{2}}-\alpha \Big )$ and $dz=(\alpha +\beta x)dx$. Using these values in Eq. (10), we will get

11 $$\begin{aligned} \mu ^{'}_{r}=\frac{\big (p-1\big )}{\beta ^r\ln \big (p\big )}\int _{0}^{\infty }\frac{\Big (\big (\alpha ^2+2\beta z\big )^{\frac{1}{2}}-\alpha \Big )^{r} e^{-z}}{\Big (1-(1-p)e^{-z}\Big )} dz. \end{aligned}$$

Further, using the geometric sum series expansion $(1-y)^{-1}=\sum \limits _{j=0}^{\infty } y^{j}$ where $|y|<1$, we can write

12 $$\begin{aligned} (1-(1-p)e^{-z})^{-1}=\sum \limits _{j=0}^{\infty }(1-p)^j e^{-zj}, \end{aligned}$$

The geometric series expansion provided in (12) converges under the condition $|(1-p)e^{-z}|<1.$ Since $e^{-z} \in (0, 1)$ for $z>0$, the above condition holds whenever $0<p<1$. Therefore, for $z>0$ and $0<p<1$, we have $0<(1-p)e^{-z}<1$, which ensures the convergence of the series. Similarly, using Binomial expansion $$(a+b)^n=\sum _{i=0}^{n} \begin{pmatrix} n \\ i \end{pmatrix} a^{n-i} b^{i}$$, we have

13 $$\begin{aligned} \Big (\big (\alpha ^2+2\beta z\big )^{\frac{1}{2}}-\alpha \Big )^{r}= \sum \limits _{i=0}^{r}(-1)^i \begin{pmatrix} r \\ i \end{pmatrix} \alpha ^i (\alpha ^2 + 2 \beta z)^{\frac{r-i}{2}}. \end{aligned}$$

Substituting Eqs. (12) and (13) into equation (11) will give

14 $$\begin{aligned} \mu ^{'}_{r}= & \frac{\big (p-1\big )}{\beta ^r \ln \big (p\big )} \sum \limits _{i=0}^{r}(-1)^i \begin{pmatrix} r \\ i \end{pmatrix} \alpha ^i (\alpha ^2 + 2 \beta z)^{\frac{r-i}{2}} \sum \limits _{j=0}^{\infty }(1-p)^j \int \limits _{0}^{\infty }e^{-z(j+1)}dz. \end{aligned}$$

For further simplification, recall the Binomial expansion $$(\alpha ^2 +2 \beta z)^{\frac{r-i}{2}}=\sum \limits _{k=0}^{\infty } \begin{pmatrix} \frac{r-i}{2} \\ k \end{pmatrix} \alpha ^{r-i-2k} 2^{k} \beta ^{k} z^{k}$$. Let the general term of the series be $$T_{k}= \begin{pmatrix} \frac{r-i}{2} \\ k \end{pmatrix} \alpha ^{r-i-2k} 2^{k} \beta ^{k} z^{k}$$. To examine convergence, consider the ratio of successive terms $\Big |\frac{T_{k+1}}{T_{k}}\Big |=\Big |\frac{\frac{r-i}{2}-k}{k+1}\Big | \frac{2\beta z}{\alpha ^2}$. Taking the limit as $k \rightarrow \infty$, we obtain $\lim _{k \rightarrow \infty }\Big |\frac{T_{k+1}}{T_{k}}\Big |= \frac{2\beta z}{\alpha ^2}$, since $\Big |\frac{\frac{r-i}{2}-k}{k+1}\Big | \Rightarrow 1$. By the ratio test, the series converges absolutely whenever $2 \beta z < \alpha ^2$. Now, substitute this Binomial expansion in Eq. (14) will give

15 $$\begin{aligned} \mu ^{'}_{r}= & \frac{(1-p)}{\ln (p)}\sum \limits _{i=0}^r \sum \limits _{j=0}^{\infty }\sum \limits _{k=0}^{\infty }(-1)^{i} \begin{pmatrix} r \\ i \end{pmatrix} \begin{pmatrix} \frac{r-i}{2} \\ k \end{pmatrix} \alpha ^{r-2k} (1-p)^j 2^{k} \beta ^{k-r} \int _{0}^{\infty } z^{k} e^{-z(j+1)}dz \nonumber \\= & \frac{(1-p)}{\ln (p)}\sum \limits _{i=0}^r \sum \limits _{j=0}^{\infty }\sum \limits _{k=0}^{\infty }(-1)^{i} \begin{pmatrix} r \\ i \end{pmatrix} \begin{pmatrix} \frac{r-i}{2} \\ k \end{pmatrix} \alpha ^{r-2k} (1-p)^j 2^{k} \beta ^{k-r} \frac{\Gamma (k+1)}{(j+1)^{k+1}} \end{aligned}$$

where $\Gamma (a)=\int \limits _{0}^{\infty } t^{a-1}e^{-t} dt$ represents the ordinary gamma function, and the convergence of the above series expansion is ensured under the conditions $0<p<1$ and $2 \beta z < \alpha ^2$.

Further, the mean, variance $(\sigma ^2)$, skewness $(\gamma _{1})$ and kurtosis $(\gamma _{2})$ of the Log-LFR distribution are derived using the first four moments. The detailed expressions for these measures are provided below for clarity and reference:

$$\mu '_1 = E[X]$$, $$\sigma ^2 = \mu '_2 - (\mu '_1)^2$$, $$\gamma _{1} =\frac{\mu '_{3}-3\mu '_{1}\mu '_{2}+2{\mu '_{1}}^3}{(\mu '_{2}-({\mu '_{1}}^2))^\frac{3}{2}}$$, $$\gamma _{2} = \frac{\mu '_{4}-4\mu '_{3}\mu '_{1}+6\mu '_{2}(\mu '_{1})^2-3(\mu '_{1})^4}{(\mu '_{2}-\mu '_{1})^2}$$.

**Table 1:** The numerical values of first four moments and other associated measures of the Log-LFR distribution for different values of $\alpha$, $\beta$ and *p*.

$(\alpha ,\beta )$	*p*	$\mu _1$	$\mu _2$	$\mu _3$	$\mu _4$	$\sigma ^2$	$\gamma _1$	$\gamma _2$
(0.5, 1.5)	0.3	0.6040	0.5822	0.7258	1.0770	0.2173	1.1011	4.1992
	0.5	0.6670	0.6752	0.8646	1.3027	0.2302	0.9682	3.8576
	0.9	0.7452	0.8018	1.0650	1.6417	0.2464	0.8182	3.5169
(0.8, 2.8)	0.3	0.4672	0.3648	0.3749	0.4623	0.1465	1.2026	4.4959
	0.5	0.5185	0.4251	0.4482	0.5607	0.1563	1.0620	4.0957
	0.9	0.5824	0.5079	0.5550	0.7095	0.1686	0.9037	3.6965
(1.8, 0.2)	0.3	0.3912	0.3451	0.4698	0.8467	0.1920	2.1923	9.7069
	0.5	0.4437	0.4116	0.5722	1.0408	0.2146	2.0006	8.5741
	0.9	0.5128	0.5082	0.7290	1.3466	0.2452	1.7952	7.4103

Table 1 presents the first four moments, variance, skewness and kurtosis of the Log-LFR distribution for three different parameter settings. The findings show that as *p* increases, both the mean and variance rise, while skewness and kurtosis decline, resulting in distributions that are less skewed and have lighter tails. In all cases the distributions remain positively skewed with heavier-than-normal tails. Among the parameter settings, (1.8, 0.2) produces the greatest skewness and kurtosis, whereas (0.8, 2.8) yields the lowest, illustrating the flexibility of the parameter *p* in shaping distributional characteristics for lifetime data.

### Moment generating function

The moment generating function (MGF) is an essential tool in the analysis of random variables due to its ability to accurately capture the characteristics of the distribution. It offers a systematic and accurate approach to calculating the moments of a random variable, which are necessary for better understanding its central tendency, dispersion, and higher-order properties. In addition, the MGF is a potent tool for both theoretical investigations and practical applications in probability and statistics, as it uniquely determines the probability distribution of a random variable under specific regularity conditions. If X has the Log-LFR distribution, then the MGF (denoted as $M_X(t)$) is given by

16 $$\begin{aligned} M_X(t)= E(e^{tx})=\sum \limits _{r=0}^{\infty }\frac{t^{r}}{r!} \mu ^{'}_{r}. \end{aligned}$$

By substituting the expression for $\mu ^{'}_{r}$ from Eq. (15) into Eq. (16), we obtain the final form of the MGF as follows:

$$\begin{aligned} M_X(t)&=\frac{(p-1)}{\ln (p)}\sum \limits _{r=0}^{\infty }\sum \limits _{i=0}^r\sum \limits _{j=0}^\infty \sum \limits _{k=0}^{\infty } \frac{t^r}{r!}(-1)^{i} \begin{pmatrix} r \\ i \end{pmatrix} \begin{pmatrix} \frac{r-i}{2} \\ k \end{pmatrix} (1-p)^j \alpha ^{r-2k} 2^{k} {\beta ^{k-r}} \frac{\Gamma (k+1)}{(j+1)^{k+1}}. \end{aligned}$$

### Quantile function

The quantile function is vital in distribution modeling, as it allows for the generation of random variates and provide direct insights into the distribution’s spread and behavior, both in theoretical and applied modeling contexts. The quantile function of any distribution is defined as $Q(u) = F^{-1}(u)$ where *Q*(*u*) is the quantile function corresponding to the cumulative distribution function *F*(*x*) for $0< u < 1$. By taking the cdf of the Log-LFR distribution and inverting it accordingly, the quantile function is obtained as follows:

17 $$\begin{aligned} Q_{Log-LFR}(u)=\beta ^{-1}\Big [\Big (\alpha ^2-2 \beta \ln \Big (\frac{1-p^{(1-u)}}{{1-p}}\Big )\Big )^{\frac{1}{2}}- \alpha \Big ], ~0<u<1,~ \alpha , \beta>0. \end{aligned}$$

### Reliability measures

In reliability analysis, the hazard rate, reversed hazard rate and mean residual life functions play a crucial role in characterizing the lifetime behavior of a system or component. The hazard rate function quantifies the instantaneous failure rate at any given time, providing insight into the aging properties of a distribution such as whether the failure rate increases (indicating wear-out), decreases (indicating early failures) or remains constant. The reversed hazard rate function is particularly useful in modeling scenarios where backward recurrence times or the likelihood of survival from the past are of interest. The mean residual life function is useful tool for maintenance scheduling up to a specific period. These reliability measures provide a comprehensive framework for analyzing failure mechanisms, guiding system design improvements, and optimizing maintenance strategies. The hazard rate, reversed hazard rate and mean residual life functions of the Log-LFR distribution are respectively given by 3

18 $$\begin{aligned} h_{Log-LFR}(x)=\frac{(p-1)(\alpha +\beta x)e^{-\big (\alpha x+\frac{\beta }{2} x^2\big )}}{\Big (1-(1-p)e^{-\big (\alpha x +\frac{\beta }{2} x^2\big )}\Big )\ln \Big (1-(1-p)e^{-\big (\alpha x +\frac{\beta }{2} x^2\big )}\Big )}, \end{aligned}$$

19 $$\begin{aligned} r_{Log-LFR}(x)=\frac{(p-1)(\alpha +\beta x)e^{-\big (\alpha x+\frac{\beta }{2}x^2\big )}}{\left( 1-(1-p)e^{-\big (\alpha x+\frac{\beta }{2}x^2\big )}\right) \ln \left( {\frac{p}{\Big (1-(1-p)e^{-\big (\alpha x +\frac{\beta }{2}x^2\big )}\Big )}}\right) }, \end{aligned}$$

20 $$\begin{aligned} \text{ and }~~m_{Log-LFR}(x)=\left( \frac{(p-1) (\ln (p))^{2}}{\ln \big (-(1-p)e^{-(\alpha x+\frac{\beta }{2}x^2)}\big )}\sum \limits _{j=0}^\infty \left( \frac{(x(j+1)+1)(1-p)^j e^{-x(j+1)}}{(j+1)^{2}}\right) \right) -x \end{aligned}$$

where $$x>0$$ and $$\alpha , \beta , p>0.$$

**Fig. 3:**
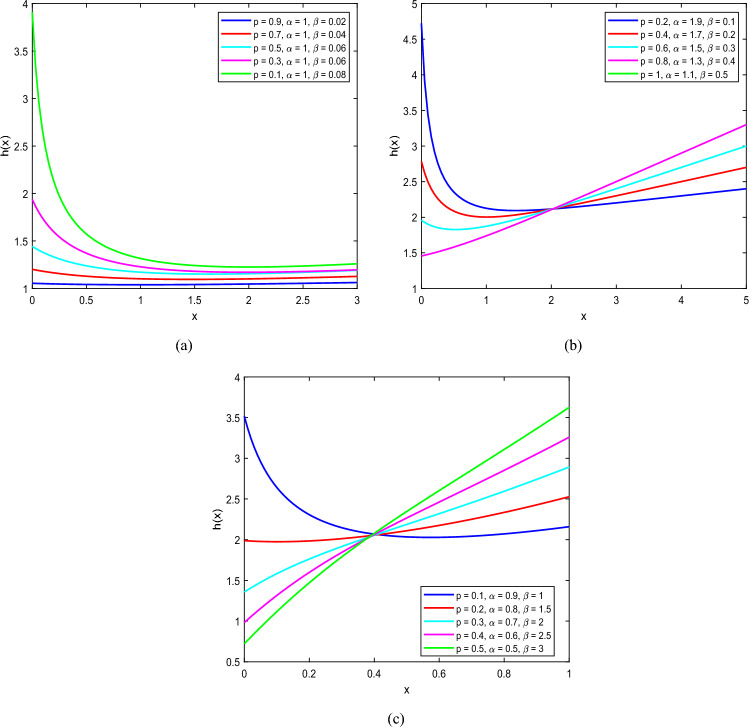
The hrf plots of the log-LFR distribution for different parameter values. It takes different shapes such as constant, increasing, decreasing, and bathtub shape.

The various shapes of the hrf of the Log-LFR distribution are given in Fig. 3.

### Rènyi entropy

The concept of entropy plays an essential role in information theory. The Rènyi entropy (see Rènyi^20^) is a one parameter generalization of entropy measure. It is used as indices of diversity and quantify the randomness or uncertainty of a system. The Rènyi entropy of the Log-LFR distribution is defined as

21 $$\begin{aligned} {R}_{Log-LFR}(\gamma )=\frac{1}{1-\gamma } \ln \Big ({{\int \limits _{0}^\infty f_{Log-LFR}^{\gamma }(x)dx}}\Big ), ~~\gamma (\ne 1)>0. \end{aligned}$$

To get the closed form expression of Eq. (21), we need to solve the integration term by substituting the pdf of the Log-LFR distribution given in Eq. (9) as follows:

22 $$\begin{aligned} {\int \limits _{0}^\infty f_{Log-LFR}^{\gamma }(x)dx}=\Big (\frac{p-1}{\ln (p)}\Big )^{\gamma }\int \limits _{0}^\infty \frac{(\alpha +\beta x)^{\gamma }e^{-\gamma \big (\alpha x+\frac{\beta }{2}x^2\big )}}{\Big (1-(1-p)e^{-\big (\alpha x+\frac{\beta }{2}x^2\big )}\Big )^{\gamma }}dx. \end{aligned}$$

Now, set $z=(\alpha x+\frac{\beta }{2}x^2)$, which implies that $x=\frac{(\alpha ^2 + 2 \beta z)^{1/2}-\alpha }{\beta }$ and $dz=(\alpha +\beta x) dx$. After substitution of these values, we get

23 $$\begin{aligned} {\int \limits _{0}^\infty f_{Log-LFR}^{\gamma }(x)dx}=\Big (\frac{p-1}{\ln (p)}\Big )^{\gamma } \int _0^{\infty } \frac{(\alpha ^{2} + 2 \beta z)^\frac{\gamma -1}{2} e^{-z{\gamma }}}{\left( {1-(1-p)e^{-z}}\right) ^{\gamma }} dz. \end{aligned}$$

Using the generalized binomial expansions, $$(1-(1-p)e^{-z})^{-\gamma }=\sum \limits _{k=0}^{\infty } \begin{pmatrix} k+\gamma -1 \\ k \end{pmatrix} (1-p)^{k}e^{-zk}$$ and

$$(\alpha ^2 + 2\beta x^2)^\frac{\gamma -1}{2} =\sum \limits _{i=0}^{\infty } \begin{pmatrix} \frac{\gamma -1}{2}\\ i \end{pmatrix} \alpha ^{\frac{\gamma -1-2i}{2}} (2\beta z)^{i}$$, Eq. (23) can be written as

24 $$\begin{aligned} {\int \limits _{0}^\infty f_{Log-LFR}^{\gamma }(x)dx}&=\Big (\frac{p-1}{\ln (p)}\Big )^{\gamma } \sum \limits _{i=0}^{\infty }\sum \limits _{k=0}^{\infty } \begin{pmatrix} \frac{\gamma -1}{2}\\ i \end{pmatrix} \begin{pmatrix} \frac{k+\gamma -1}{2}\\ k \end{pmatrix} \alpha ^{\frac{\gamma -1-2i}{2}} (2\beta )^{i}(1-p)^{k} \int _{0}^{\infty } z^{i} e^{-z(\gamma +k)}dz \nonumber \\&=\Big (\frac{p-1}{\ln (p)}\Big )^{\gamma } \sum \limits _{i=0}^{\infty }\sum \limits _{k=0}^{\infty } \begin{pmatrix} \frac{\gamma -1}{2}\\ i \end{pmatrix} \begin{pmatrix} \frac{k+\gamma -1}{2}\\ k \end{pmatrix} \alpha ^{\frac{\gamma -1-2i}{2}} (2\beta )^{i}(1-p)^{k} \frac{\Gamma (i+1)}{(\gamma +k)^{i+1}}. \end{aligned}$$

After evaluated the integral by using the gamma function and substituting Eq. (24) in Eq. (21), then the Rènyi entropy of the Log-LFR distribution can be obtained as

25 $$\begin{aligned} \begin{aligned} R_{Log\text {-}LFR}(\gamma )&= \frac{1}{1-\gamma } \Bigg \{ \ln \left( \left( \frac{p-1}{\ln (p)}\right) ^{\gamma } \right) \\&\quad + \ln \Bigg ( \sum _{i=0}^{\infty }\sum _{k=0}^{\infty } \left( {\begin{array}{c}\tfrac{\gamma -1}{2}\\ i\end{array}}\right) \left( {\begin{array}{c}\tfrac{k+\gamma -1}{2}\\ k\end{array}}\right) \alpha ^{\tfrac{\gamma -1-2i}{2}} (2\beta )^{i} (1-p)^{k} \frac{\Gamma (i+1)}{(\gamma +k)^{i+1}} \Bigg ) \Bigg \}. \end{aligned} \end{aligned}$$

### Distribution of order statistics

The analysis of reliability and life testing can uncover a variety of applications for order statistics. These statistics can characterize the longevity of a reliability system for specific units or components. Suppose $X_{1:n}\le X_{2:n}\le ...\le X_{n:n}$ denotes the order statistics of a random sample $X_{1},X_{2},...,X_{n}$ drawn from a distribution function *F*(*x*) with pdf *f*(*x*). Thus, the pdf of *i*-th order statistics (denoted as $X_{i:n}$) is given by

26 $$\begin{aligned} f_{X_{i:n}}(x)=\frac{n!}{(i-1)!(n-i)!} f(x) (F(x))^{i-1}(1-F(x))^{n-i}, ~i=1,2,...,n. \end{aligned}$$

This formula allows for the calculation of the pdf of any specific order statistic in a sample from a continuous population. Now, substituting the pdf and cdf of the Log-LFR distribution correspondingly provided in Eqs. (8) and (9), into Eq.(26) will give the pdf of *i*-th order statistics $X_{i:n}$ as mentioned below:

27 $$\begin{aligned} f_{X_{i:n}}(x)= & \frac{n!}{(i-1)!(n-i)!}{\frac{(p-1)(\alpha +\beta x)e^{-\big (\alpha x +\frac{\beta }{2} x^2\big )}}{\Big (1-(1-p)e^{-\big (\alpha x +\frac{\beta }{2} x^2\big )}\Big )\ln (p)}} \nonumber \\ & \times \left\{ {{1-\frac{\ln \Big (1-(1-p)e^{-\big (\alpha x +\frac{\beta }{2} x^2\big )}\Big )}{\ln (p)}}}\right\} ^{i-1} \left\{ {\frac{\ln \Big (1-(1-p)e^{-\big (\alpha x +\frac{\beta }{2} x^2\big )}\Big )}{\ln (p)}}\right\} ^{n-i}. \end{aligned}$$

Therefore, the pdf of the largest order statistic $X_{n:n}$ and the smallest order statistic $X_{1:n}$ are respectively given by

$$\begin{aligned} f_{X_{n:n}}(x)=n{\frac{(p-1)(\alpha +\beta x)e^{-\big (\alpha x +\frac{\beta }{2} x^2\big )}}{\Big (1-(1-p)e^{-\big (\alpha x +\frac{\beta }{2} x^2\big )}\Big )\ln (p)}} \left\{ {1-\frac{\ln \Big (1-(1-p)e^{-\big (\alpha x +\frac{\beta }{2} x^2\big )}\Big )}{ln(p)}}\right\} ^{n-1} \end{aligned}$$

$$\begin{aligned} \text{ and }~~f_{X_{1:n}}(x)=n{\frac{(p-1)\big (\alpha +\beta x\big )e^{-\big (\alpha x +\frac{\beta }{2} x^2\big )}}{\Big (1-(1-p)e^{-\big (\alpha x +\frac{\beta }{2} x^2\big )}\Big )\ln (p)}} \left\{ {\frac{\ln \Big (1-(1-p)e^{-\big (\alpha x +\frac{\beta }{2} x^2\big )}\Big )}{ln(p)}}\right\} ^{n-1}. \end{aligned}$$

The study of the smallest and largest order statistics is fundamental in the development and evaluation of probability distribution models, particularly in the context of reliability engineering. The smallest order statistic corresponds to the time to first failure and is directly associated with the reliability of series systems, where system failure occurs upon the failure of the first component. Conversely, the largest order statistic reflects the time to last failure and is crucial in analyzing parallel systems, which fail only when all components have failed. Understanding the behavior of these extreme values under the proposed distribution provides critical insights into system robustness, failure dynamics, and performance evaluation. Therefore, incorporating the analysis of order statistics significantly enhances the practical relevance and applicability of the new distribution in real-world reliability and risk assessment scenarios.

## Stochastic orderings

The concept of stochastic ordering is a powerful tool in reliability theory and statistical inference, as it allows for the comparison of random variables or systems in terms of their likelihood of yielding larger or smaller values. Such comparisons are especially useful in assessing the relative reliability, risk or performance of components or systems without relying on specific numerical values. In the context of the Log-LFR distribution, stochastic ordering provides a framework to determine under what conditions one system is more reliable than another. Specifically, when two systems follow the Log-LFR distributions with different parameter sets, their lifetimes can be stochastically ordered based on certain inequalities involving the shape and scale parameters. In the following theorem, we formally establish the conditions under which such an ordering holds, thereby demonstrating how parameter variations influence the relative behavior of system components governed by the Log-LFR model. Before presenting the theorem, we provide the definition of usual stochastic ordering as utilized in the theorem, proposed by Shaked and Shanthikumar^16^.

### Definition 1

Let *X* and *Y* be two non-negative random variables having survival functions $\bar{F}_X(x)$ and $\bar{F}_Y(x)$, respectively.

(i) *X* is stated to be smaller than *Y* in the sense of the usual stochastic (st) ordering $(X\leqslant _{st}Y)$, if $\bar{F}_X(x) \le \bar{F}_Y(x)$, for all $x\in \mathbb {R^+}.$
(ii) *X* is stated to be smaller than *Y* in the sense of the likelihood ratio ordering (lr) ordering $(X\leqslant _{lr}Y)$ if $\frac{f_{Y}(x)}{f_{X}(x)}$ is increasing in *x* for all $x\in \mathbb {R^+}.$
(iii) *X* is stated to be smaller than *Y* in the sense of the hazard rate ordering (hr) ordering $(X\leqslant _{hr}Y)$, if $h_{X}(x)\le h_{Y}(x)$ for all $x\in \mathbb {R^+}.$

### Theorem 1

*Consider two independent random variables* *X* *and* *Y*, *where* *X* *follows a log-LFR distribution with parameters* $\alpha$, $\beta$, $p_{1}$*; and Y follows a Log-LFR distribution with parameters* $\alpha$, $\beta$, $p_{2}$*; respectively, and for* $p_{2}\ge p_{1}$ *then* $X \le _{lr} Y$.

**Proof:** From Eq. (9), we have

28 $$\begin{aligned} \frac{f_{Y}(x)}{f_{X}(x)}=\frac{(p_{2}-1)(1-(1-p_{1})e^{-(\alpha x+\frac{\beta }{2}x^2)})\ln (p_{1})}{(p_{1}-1)(1-(1-p_{2})e^{-(\alpha x+\frac{\beta }{2}x^2)})\ln (p_{2})}. \end{aligned}$$

Proceeding by taking $\ln$ of Eq. (28) on both sides and differentiate the resulting expression with respect to *x* gives us:

$$\begin{aligned} \frac{d}{dx}\ln \left( \frac{f_{Y}(x)}{f_{X}(x)}\right) =\frac{(1-p_{1})(\alpha +\beta x)e^{-(\alpha x+\frac{\beta }{2}x^2)}}{(1-(1-p_{1})e^{-(\alpha x+\frac{\beta }{2}x^2)})}-\frac{(1-p_{2})(\alpha +\beta x)e^{-(\alpha x+\frac{\beta }{2}x^2)}}{(1-(1-p_{2})e^{-(\alpha x+\frac{\beta }{2}x^2)})}. \end{aligned}$$

Thus, $\frac{d}{dx}\ln \left( \frac{f_{Y}(x)}{f_{X}(x)}\right) \ge 0$ provided that $p_{1}\le p_{2}$. Therefore, the desired ordering obtained.

### Remark 1

The following result is well known in the literature (Shaked and Shanthikumar^16^):

$$X \le _{lr} Y \Rightarrow X \le _{hr} Y \Rightarrow X \le _{st} Y.$$

The relationship between these orderings follows a cascading pattern: likelihood ratio ordering establishes hazard rate ordering, which in turn generates the usual stochastic ordering. Consequently, under the conditions stated in Theorem 1, the hazard rate and usual stochastic naturally follow from the likelihood ratio ordering.

The research is further expanded to encompass usual stochastic ordering through the examination of independent random variables $X_{i}$ and $Y_{i}$ drawn from their corresponding populations. By utilizing the series representation of the probability density function, we derive a Theorem 2 that defines the stochastic ordering relationship $X_{i} \le _{st} Y_{i}$. For comparing random variables while guaranteeing the broad applicability of findings across various reliability engineering and survival analysis contexts, these techniques provide a clear and more general configuration framework. The implementation of series expansion not only streamlines the mathematical analysis but also provides valuable understanding of how these distributions behave in comparison to one another. In the following, a stochastic comparison between series systems is established under certain sufficient conditions. These conditions enable us to characterize the ordering behavior of the system lifetimes and provide theoretical insights into their reliability performance. The formal result is presented in the theorem below.

### Theorem 2

*Let* $(X_1, X_2, \ldots , X_n)$ *and* $(Y_1, Y_2, \ldots , Y_n)$ *be two sets of random variables, where each* $X_i$ *follows a Log-LFR distribution with parameters* $\alpha _i$ *and* $\beta _i$*, and each* $Y_i$ *follows a Log-LFR distribution with parameters* $\alpha _i^*$ *and* $\beta _i^*$*, for* $i = 1, 2, \ldots , n$*, respectively. If* $\alpha _i\le \alpha ^*_i$ *and* $\beta _i\le \beta ^*_i$*, then* $X_{1:n}\leqslant _{st}Y_{1:n}$.

**Proof:** The cumulative distribution functions of $X_{1:n}$ and $Y_{1:n}$ are correspondingly defined as

29 $$\begin{aligned} F_{X_{1:n}}(x)=1-\prod \limits _{i=1}^n \left( \frac{\ln \Big (1-(1-p)e^{-(\alpha _i x +\frac{\beta _i}{2}x^2)}\Big )}{\ln (p)}\right) \end{aligned}$$

30 $$\begin{aligned} \text{ and }~~F_{Y_{1:n}}(x)=1-\prod \limits _{i=1}^n \left( \frac{\ln \Big (1-(1-p)e^{-(\alpha ^*_i x +\frac{\beta ^*_i}{2}x^2)}\Big )}{\ln (p)}\right) \end{aligned}$$

Using Definition 1 of usual stochastic ordering, we need to prove that $\bar{F}_{X_{1:n}}(x)\le \bar{F}_{Y_{1:n}}(x)$. Thus, to show

31 $$\begin{aligned} 1-\prod \limits _{i=1}^n \left( \frac{\ln \Big (1-(1-p)e^{-(\alpha _i x +\frac{\beta _i}{2}x^2)}\Big )}{\ln (p)}\right) \le 1-\prod \limits _{i=1}^n \left( \frac{\ln \Big (1-(1-p)e^{-(\alpha ^*_i x +\frac{\beta ^*_i}{2}x^2)}\Big )}{\ln (p)}\right) . \end{aligned}$$

Further, simplification of (31) will give the inequality

32 $$\begin{aligned} \alpha _i x + \frac{\beta _i}{2}x^2\le \alpha ^*_i x + \frac{\beta ^*_i}{2}x^2. \end{aligned}$$

Under the condition $\alpha _i \le \alpha ^*_i$ and $\beta _i \le \beta ^*_i$, the above inequality (32) holds. Therefore, we conclude that $X_{1:n}\le Y_{1:n}$. This completes the proof.

To illustrate the result presented in Theorem 1 and 2, we consider the following example and counterexample. Before proceeding, it is important to address a graphical challenge due to the domain of the cumulative distribution function being $x \in \mathbb {R}^+$, which makes direct visualization difficult. A practical approach to overcome this is to apply a transformation that maps the variable *x* to a new variable *y* defined over the interval (0, 1). Specifically, we employ the transformation $x = \log \left( \frac{1}{1 - y}\right)$, for $y \in (0, 1)$. This mapping facilitates a more effective graphical representation of the function by converting the infinite domain $\mathbb {R}^+$ into a finite and manageable interval. Such a transformation retains the essential characteristics of the original function while enabling easier interpretation and comparison of its behavior. The following is an example of the verification of Theorem 1 and 2 and also present counterexamples that highlight the importance of the conditions outlined in the theorems for accurate results.

### Example 1

**Fig. 4:**
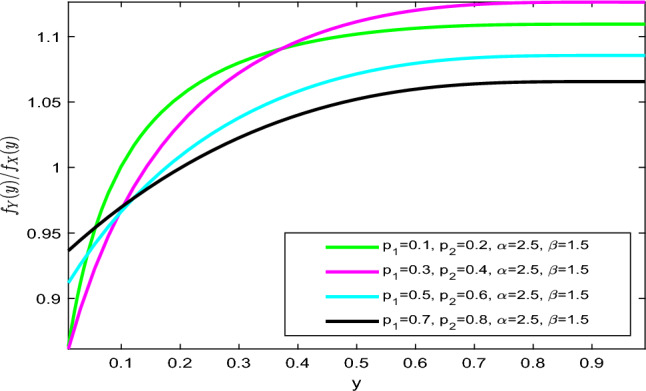
Plot of $f_{Y}(y)/f_{X}(y)$ where $y \in (0, 1)$.

Consider two random variables *X* and *Y* as mentioned in Theorem 1. For the specific values like $p_{1} = (0.01, 0.03, 0.05)$ and $p_{2} = (1.0, 3.0, 4.5)$, one can easily check that $p_{2}\ge p_{1}$. From the graphical view, it can be observer that the likelihood ratio function $\frac{f_{Y}(y)}{f_{X}(y)}$ is increasing in *y*, demonstrating that $X \le _{lr} Y$. Figure 4 illustrates Theorem 1, confirming that for fixed parameters $\alpha$ and $\beta$ and varying parameter *p*, the likelihood ratio ordering result holds.

### Example 2

**Fig. 5:**
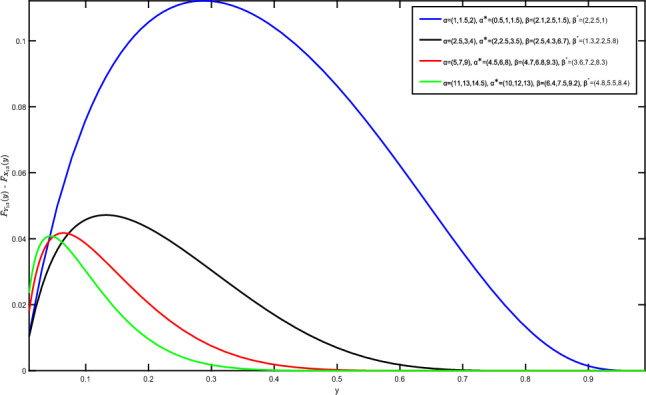
(a): Plot of the difference $\bar{F}_{Y_{1:3}}(y)-\bar{F}_{X_{1:3}}(y)$ where $y \in (0, 1)$.

Let $(X_{1},X_{2},X_{3})$ and $(Y_{1},Y_{2},Y_{3})$ be two sets of independent random variables such that each $X_i$ follows the Log-LFR distribution with parameters $\alpha _i$ and $\beta _i$, and each $Y_i$ follows the Log-LFR distribution with parameters $\alpha ^*_i$ and $\beta ^*_i$, for $i = 1, 2, 3$. To validate the result established in Theorem 2, different sets of values for the parameter vectors $(\alpha _1, \alpha _2, \alpha _3), (\alpha ^*_1, \alpha ^*_2, \alpha ^*_3), (\beta _1, \beta _2, \beta _3)$ and $(\beta ^*_1, \beta ^*_2, \beta ^*_3)$ are considered for a fixed value of $p> 0$. The plot of the difference $\bar{F}_{Y_{1:3}}(y) - \bar{F}_{X_{1:3}}(y)$ is presented in Fig. 5 for various parameter configurations to visually demonstrate the stochastic ordering.

### Counterexample 1

**Fig. 6:**
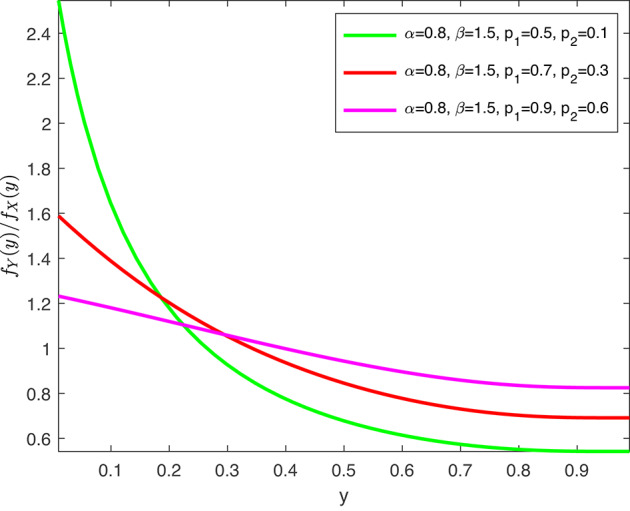
Plot of $f_{Y}(y)/f_{X}(y)$ where $y \in (0, 1)$.

Assume $p_{1} = (0.5, 0.7, 0.9)$ and $p_{2} = (0.1, 0.3, 0.6)$. This violate the condition mentioned in Theorem 1 by having $p_{1} \ge p_{2}$. Thus, the likelihood ratio ordering, that is, $X \le _{lr} Y$ fails to hold. In this case, the likelihood ratio function $\frac{f_{Y}(y)}{f_{X}(y)}$ is not monotonically increasing in *y*, violating the requirement for likelihood ratio ordering. Figure 6 demonstrates that the parameter condition $p_{2} \ge p_{1}$ is essential for establishing the stochastic ordering relationship between Log-LFR distributions.

### Counterexample 2

**Fig. 7:**
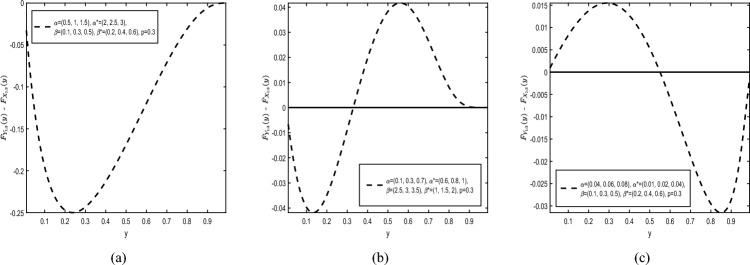
(**a**): represents the plot of $\bar{F}_{Y_{1:3}}(y)-\bar{F}_{X_{1:3}}(y)$ where $y\in \mathbb (0,1)$ with conditions $\alpha _i < \alpha ^*_i$, $\beta _i < \beta ^*_i$. (**b**): represents the plot of $\bar{F}_{Y_{1:3}}(y)-\bar{F}_{X_{1:3}}(y)$ where $y\in \mathbb (0,1)$ with conditions $\alpha _i < \alpha ^*_i$, $\beta _i> \beta ^*_i$. (**c**): represents the plot of $\bar{F}_{Y_{1:3}}(y)-\bar{F}_{X_{1:3}}(y)$ where $y\in \mathbb (0,1)$ with conditions $\alpha _i> \alpha ^*_i$, $\beta _i < \beta ^*_i$.

Under the setup in Example 2, the stochastic ordering ${X_{1:3}} \le {Y_{1:3}}$ may not hold if the conditions $a_{i} \le a^*_{i}$ and $b_{i} \le b^*_{i}$ are violated for any $i = 1, 2, 3$. Figure 7 demonstrates this through different values of parameters where $\bar{F}_{Y_{1:3}}(y)-\bar{F}_{X_{1:3}}(y)$ becomes negative for some $y\in (0,1)$, demonstrating that the ordering fails under these conditions.

## Estimation and simulation study

This section addresses the parameter estimation of the Log-LFR distribution using the maximum likelihood estimation approach. Given the non-linear nature of the likelihood equations, numerical optimization methods are applied to compute the estimates. To assess the performance of these estimators, a Monte Carlo simulation study is carried out.

### Maximum likelihood estimation

In this section, we examine the conventional approaches used to estimate the parameter of the Log-LFR distribution. These estimation methods involve optimizing an objective function by maximization or minimization, intending to obtain the most appropriate estimate. Let $x_{1},x_{2},...,x_{n}$ be the observations of a random sample of size *n* of the Log-LFR distribution. Then, the estimation of the parameters for the Log-LFR distribution can be done using the maximum likelihood estimation, where the objective function is to maximize, for $\alpha$, $\beta$ and *p*, the following likelihood function is given by

33 $$\begin{aligned} l=\prod _{i=1}^n f_{i}(x)=(p-1)^n \Big (\frac{1}{\ln p}\Big )^n\prod _{i=1}^n\frac{\big (\alpha +\beta x_{i}\big )e^{-\big (\alpha x_{i}+\frac{\beta }{2}x_{i}^2\big )}}{\Big [1-(1-p)e^{-\big (\alpha x_{i}+\frac{\beta }{2}x_{i}^2\big )}\Big ]\ln p}. \end{aligned}$$

Now, the log-likelihood function $L=\ln l$ can be defined as

34 $$\begin{aligned} L=n\ln \Big (\frac{p-1}{\ln p}\Big )+\sum \limits _{i=1}^n\ln (\alpha +\beta x_{i})-\sum \limits _{i=1}^n\big (\alpha +\beta x_{i}\big )e^{-\big (\alpha x_{i}+\frac{\beta }{2}x_{i}^2\big )}-\sum \limits _{i=1}^n\ln \Big [1-(1-p)e^{-\big (\alpha x_{i}+\frac{\beta }{2}x_{i}^2\big )}\Big ]. \end{aligned}$$

The resulting first-order partial derivatives of the previous Eq. (34) with respective parameters are given as follows:

35 $$\begin{aligned} \frac{\partial L}{\partial \alpha }= & \sum \limits _{i=1}^n\frac{1}{(\alpha +\beta x_{i})}-\sum \limits _{i=1}^n x_{i}-(1-p)\sum \limits _{i=1}^n\frac{x_{i}e^{-(\alpha x_{i}+\frac{\beta }{2}x_{i}^2)}}{\Big [1-(1-p)e^{-(\alpha x_{i}+\frac{\beta }{2}x_{i}^2)}\Big ]},\end{aligned}$$

36 $$\begin{aligned} \frac{\partial L}{\partial \beta }= & \sum \limits _{i=1}^n\frac{x_{i}}{(\alpha +\beta x_{i})}-\frac{1}{2}\sum \limits _{i=1}^n x_{i}^2 -\frac{(1-p)}{2}\sum \limits _{i=1}^n\frac{x_{i}^2e^{-(\alpha x_{i}+\frac{\beta }{2}x_{i}^2)}}{\Big [1-(1-p)e^{-(\alpha x_{i}+\frac{\beta }{2}x_{i}^2)}\Big ]},\end{aligned}$$

37 $$\begin{aligned} \text{ and }~~\frac{\partial L}{\partial p}= & \frac{n}{(p-1)}-\frac{n}{p\ln p}-\sum \limits _{i=1}^n\frac{e^{-(\alpha x_{i}+\frac{\beta }{2}x_{i}^2)}}{\Big [1-(1-p)e^{-(\alpha x_{i}+\frac{\beta }{2}x_{i}^2)}\Big ]}. \end{aligned}$$

Maximum likelihood parameter estimation for the Log-LFR distribution involves solving the likelihood equations obtained by setting the first-order partial derivatives of the log-likelihood function to zero. Due to the analytical intractability of these equations, we resort to numerical optimization techniques. The Limited Memory Broyden-Fletcher-Goldfarb-Shanno algorithm with bound constraints (L-BFGS-B) algorithm is implemented to iteratively determine the maximum likelihood estimates under appropriate parameter constraints.

The percentile bootstrap method constructs confidence intervals through resampling the original data with replacement to create N bootstrap samples. Each sample generates parameter estimates $(\hat{\alpha }^{(b)}, \hat{\beta }^{(i)}, \hat{p}^{(i)})$ for $i= 1, 2,..., N$ using identical estimation procedures. Confidence intervals at the $100(1-\gamma )\%$ level are formed by taking the $(\gamma /2)^{\text {th}}$ and $(1-\gamma /2)^{\text {th}}$ percentiles from the ordered bootstrap estimates. This approach creates empirical distributions that directly capture parameter uncertainty without requiring distributional assumptions. The method extends to derived functions by computing reliability $\hat{R}^{(i)}(t)$ and hazard $\hat{h}^{(i)}(t)$ values for each bootstrap replication using the corresponding parameter estimates. Confidence intervals for these functions follow the same percentile-based construction from their bootstrap distributions.

### Simulation study

This section presents a detailed simulation study designed to assess the performance of the maximum likelihood estimators (MLEs) for the parameters of the Log-LFR distribution. To evaluate the effectiveness of the estimators, random samples were generated using the inversion method based on the quantile function defined in Eq. (17) of the proposed model. The simulation considered varying sample sizes $n = 50, 100, 200, 350$, with each scenario replicated 1000 times. Two parameter settings were examined: Case-I with $\alpha = 0.4$, $\beta = 0.5$, $p = 0.2$; and Case-II with $\alpha = 0.3$, $\beta = 0.8$, $p = 0.1$. The performance of the estimators was assessed based on point estimates, bias, and mean squared error (MSE). The simulation results, as summarized in Table 2, indicate that the MLEs become increasingly accurate with larger sample sizes. Specifically, the bias and MSE values for all parameters decrease steadily as *n* increases, demonstrating the consistency and efficiency of the estimators. Furthermore, the simulation investigated the accuracy of the estimators in estimating reliability and hazard rate functions at specific time points ($t = 2.5$, $t = 3$ for Case-I and Case-II),further confirming the robustness of the model. The confidence interval (CI) is reported with their Lower Confidence Point (LCP) and Upper Confidence Point (UCP).

All computations were carried out using R software. Overall, this simulation study validates the applicability of the proposed MLE method for the Log-LFR distribution and highlights its potential in producing reliable parameter estimates, thereby supporting its use in reliability analysis and survival data modeling.

**Table 2:** Estimate, Bias MSE and CI for $\alpha =0.4$, $\beta =0.5$, $p=0.2$; reliability and hazard functions at different time points.

n		Case-I				CI	
	Parameter	True value	Estimate	Bias	MSE	LCP	UCP
50	$\alpha$	0.4	0.5576	0.1576	0.3165	0.0100	1.7672
	$\beta$	0.5	0.4750	−0.0250	0.0704	0.0100	1.0146
	p	0.2	0.9623	0.7623	3.0368	0.0100	5.000
	$R(t)|_{t=2.5}$	0.0396	0.0175	0.0015	0.0001	0.0082	0.0829
	$h(t)|_{t=2.5}$	1.7037	2.0091	0.0844	0.4069	1.0813	2.9836
	$R(t)|_{t=3}$	0.0160	0.0175	0.0015	0.0001	0.0018	0.0456
	$h(t)|_{t=3}$	1.9246	2.0091	0.0844	0.4069	1.1353	3.4151
100	$\alpha$	0.4	0.5346	0.1346	0.2630	0.0126	1.6773
	$\beta$	0.5	0.4531	−0.0469	0.0520	0.0100	0.8388
	p	0.2	0.7364	0.5364	1.9303	0.0100	5.000
	$R(t)|_{t=2.5}$	0.0396	0.0170	0.0010	0.0001	0.0138	0.0699
	$h(t)|_{t=2.5}$	1.7037	1.9199	−0.0048	0.1776	1.1931	2.5747
	$R(t)|_{ t=3}$	0.0160	0.0170	0.0010	0.0001	0.0035	0.0361
	$h(t)|_{t=3}$	1.9246	1.9199	−0.0048	0.1776	1.2559	2.9316
200	$\alpha$	0.4	0.5286	0.1286	0.2245	0.0326	1.6696
	$\beta$	0.5	0.4416	−0.0584	0.0412	0.0100	0.7229
	p	0.2	0.6249	0.4249	1.2937	0.0175	4.5983
	$R(t)|_{t=2.5}$	0.0396	0.0398	0.0002	0.0001	0.0205	0.0617
	$h(t)|_{ t=2.5}$	1.7037	1.6837	−0.0200	0.0539	1.3053	2.2226
	$R(t)|_{t=3}$	0.0160	0.0169	0.0009	0.0000	0.0062	0.0304
	$h(t)|_{t=3}$	1.9246	1.8780	−0.0467	0.0874	1.3887	2.5562
350	$\alpha$	0.4	0.5223	0.1223	0.1855	0.0486	1.5498
	$\beta$	0.5	0.4402	−0.0598	0.0338	0.0100	0.6796
	p	0.2	0.5424	0.3424	0.8788	0.0220	3.6029
	$R(t)|_{t=2.5}$	0.0396	0.0397	0.0002	0.0001	0.0240	0.0571
	$h(t)|_{ t=2.5}$	1.7037	1.6732	−0.0305	0.0350	1.3529	2.0783
	$R(t)|_{t=3}$	0.0160	0.0168	0.0008	0.0000	0.0080	0.0272
	$h(t)|_{t=3}$	1.9246	1.8670	−0.0576	0.0597	1.4337	2.3671

**Table 3:** Estimate, Bias, MSE and CI for $\alpha =0.3$, $\beta =0.5$, $p=0.1$; and reliability and hazard functions at different time points.

n		Case-II				CI	
	Parameter	True value	Estimate	Bias	MSE	LCP	UCP
50	$\alpha$	0.3	0.7219	0.4219	0.6371	0.0100	2.2054
	$\beta$	0.8	0.6650	−0.1350	0.1808	0.0100	1.4773
	p	0.1	0.9808	0.8808	3.1481	0.0100	5.0000
	$R(t)|_{t=2.5}$	0.0154	0.0168	0.0014	0.0001	0.0016	0.0439
	$h(t)|_{ t=2.5}$	2.3413	2.4136	0.0723	0.5964	1.3688	4.1776
	$R(t)|_{t=3}$	0.0002	0.0007	0.0005	0.0000	0.0000	0.0041
	$h(t)|_{t=3}$	3.5008	3.3831	−0.1177	1.6948	1.5207	6.2521
100	$\alpha$	0.3	0.6817	0.3817	0.5324	0.0131	2.0650
	$\beta$	0.8	0.6390	−0.1610	0.1418	0.0100	1.2377
	p	0.1	0.7564	0.6564	2.0480	0.0100	5.0000
	$R(t)|_{t=2.5}$	0.0154	0.0164	0.0009	0.0001	0.0035	0.0350
	$h(t)|_{ t=2.5}$	2.3413	2.3086	−0.0327	0.2648	1.5128	3.5620
	$R(t)|_{t=3}$	0.0002	0.0006	0.0004	0.0000	0.0000	0.0028
	$h(t)|_{t=3}$	3.5008	3.2388	−0.2620	0.9675	1.6371	5.3029
200	$\alpha$	0.3	0.6763	0.3763	0.4795	0.0364	2.0449
	$\beta$	0.8	0.6209	−0.1791	0.1230	0.0100	1.0427
	p	0.1	0.6597	0.5597	1.4408	0.0179	4.3614
	$R(t)|_{t=2.5}$	0.0154	0.0163	0.0009	0.0000	0.0059	0.0294
	$h(t)|_{ t=2.5}$	2.3413	2.2566	−0.0847	0.1368	1.6548	3.0849
	$R(t)|_{t=3}$	0.0002	0.0005	0.0003	0.0000	0.0000	0.0018
	$h(t)|_{t=3}$	3.5008	3.1607	−0.3401	0.6681	1.7710	4.5548
350	$\alpha$	0.3	0.6704	0.3704	0.4277	0.0536	1.9483
	$\beta$	0.8	0.6180	−0.1820	0.1080	0.0100	0.9865
	p	0.1	0.5949	0.4949	1.1004	0.0211	3.5763
	$R(t)|_{t=2.5}$	0.0154	0.0162	0.0007	0.0000	0.0076	0.0261
	$h(t)|_{ t=2.5}$	2.3413	2.2428	−0.0986	0.0955	1.7236	2.8492
	$R(t)|_{t=3}$	0.0002	0.0004	0.0002	0.0000	0.0000	0.0014
	$h(t)|_{t=3}$	3.5008	3.1429	−0.3579	0.5400	1.8416	4.2597

Table 2 and Table 3 present the bias, MSE and CI values for the MLEs of the parameters $\alpha$ and $\beta$, as well as the reliability function *R*(*t*) and the hazard rate function *h*(*t*), evaluated at different time points ($t = 2.5$ and $t = 3$) under two parameter configurations (Case-I and Case-II). The results clearly demonstrate that as the sample size increases from $n = 50$ to $n = 350$, both the bias and MSE consistently decrease for all quantities considered. This trend indicates that the proposed estimators are asymptotically unbiased and consistent. The estimation of the reliability function *R*(*t*) exhibits particularly high accuracy, with the bias and MSE approaching zero for larger samples. On the other hand, the estimation of the hazard rate function *h*(*t*) shows greater variability, especially in smaller samples, but its performance also improves significantly with larger sample sizes. A comparison between the two cases suggests that the estimators under Case-I generally perform slightly better in smaller samples; however, this difference becomes negligible as the sample size increases. Overall, the simulation study supports the effectiveness and robustness of the proposed estimation method, affirming its practical applicability in moderate to large sample settings.

## Application to aircraft windshield failure and service data

This section presents the application of the Log-LFR distribution to aircraft windshield service and failure time datasets to evaluate its practical performance.

### Dataset-I (aircraft windshields failure time data)

In this section, we demonstrate the applicability of the proposed Log-LFR distribution using a real-life aircraft windshield failure time dataset. This dataset consists of 84 observed failure times for a specific aircraft windshield model. The observations represent the times at which the windshields failed, as reported in Murthy et al.^19^. The detailed failure times are presented in Table 4.

**Table 4:** Failure time of aircraft windshields data set.

0.040	1.866	2.385	3.443	0.301	1.876	2.481	3.467	0.309	1.899	2.610	3.478
0.557	1.911	2.625	3.578	0.943	1.912	2.632	3.595	1.070	1.914	2.646	3.699
1.124	1.981	2.661	3.779	1.248	2.010	2.688	3.924	1.281	2.038	2.823	4.035
1.281	2.085	2.890	4.121	1.303	2.089	2.902	4.167	1.432	2.097	2.934	4.240
1.480	2.135	2.962	4.255	1.505	2.154	2.964	4.278	1.506	2.190	3.000	4.305
1.568	2.194	3.103	4.376	1.615	2.223	3.114	4.449	1.619	2.224	3.117	4.485
1.652	2.229	3.166	4.570	1.652	2.300	3.344	4.602	1.757	2.324	3.376	4.663

To evaluate the performance of the proposed Log-LFR distribution, we compare it with other competing models like the Transmuted Linear Failure Rate (TLFR) Tian et al.^22^, LFR, Rayleigh (R) and Exponential (E) distributions. The pdfs of these distribution models are provided below:

$$\begin{aligned} f_{TLFR}(x)= & (\beta +\theta x)e^{-(\beta x +\frac{\theta }{2}x^2)}\Big (1-\lambda +2\lambda e^{-(\beta x +\frac{\theta }{2}x^2)}\Big ),~x>0,~\beta ,\theta>0,~|\lambda | \le 1; \\ f_{LFR}(x)= & (\alpha +\beta x)e^{-(\alpha x +\frac{\beta }{2}x^2)},~ x>0,~ \alpha ,\beta>0;\\ f_{R}(x)= & \frac{x}{\alpha }e^{(\frac{-x^2}{2\alpha })},~ x>0,~ \alpha>0;\\ \text{ and }~~f_{E}(x)= & \lambda e^{-\lambda x}, ~x>0,~\lambda>0. \end{aligned}$$

The measures of goodness of fit including the log-likelihood function evaluated at the MLEs, Akaike Information Criteria (AIC), Bayesian Information Criterion (BIC), Corrected Akaike Information Criteria (AICc), Hannan-Quinn Information Criterion (HQIC) and K-S are calculated to compare the fitted models. In general, lower values of these statistics indicate a better fit to the data. All necessary computations were performed using the R software.

**Table 5:** MLEs and goodness of fit values for aircraft windshields failure time data.

Distribution	MLEs	AIC	BIC	AICc	HQIC	-lnL	K-S
Log-LFR	$\hat{\alpha } =0.0306$	225.8799	233.1723	226.1799	228.8114	109.9399	0.0786
	$$\hat{\beta } =0.3326$$						
	$$\hat{p} =3.5$$						
TLFR	$\hat{\beta }=0.0767$	261.9424	269.2348	262.2424	264.8739	127.971	0.0560
	$$\hat{\theta } =0.3037$$						
	$$\hat{\lambda } =-0.7675$$						
LFR	$\hat{\alpha } =0.0107$	267.1595	272.0211	267.3076	269.1138	131.5797	0.1158
	$$\hat{\beta } =0.25$$						
Rayleigh	$\hat{\alpha } = 0.75$	311.0859	313.5167	311.1347	312.063	154.5429	0.4790
Exponential	$\hat{\lambda }=0.3910$	329.7539	334.6156	329.9021	331.7083	162.877	0.3028

**Fig. 8:**
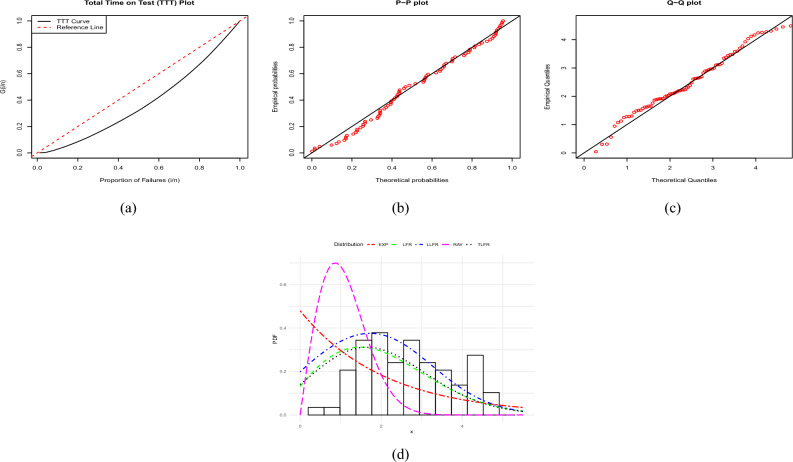
(**a**) TTT plot, (**b**) P-P plot, (**c**) Q-Q plot and (**d**) The fitted pdfs and histogram plot.

The TTT plot indicates a decreasing failure rate, suggesting that aircraft windshield failures are more likely to occur early in their lifespan. Maximum likelihood estimates (MLEs) and other statistical measures of the model parameters are presented in Table 5, which demonstrate that the Log-LFR distribution provides the best fit among several competing lifetime distributions with similar statistical characteristics. Figure 8 offers comprehensive graphical evidence supporting this conclusion through four key visualizations. The TTT plot Fig. 8(a) shows a convex shape, further confirming the early-life failure pattern. The P–P and Q–Q plots Fig. 8(b) and Fig. 8(c) display strong consistency between the empirical and theoretical values, demonstrating the model’s adequacy. Lastly, the probability density function comparison Fig. 8(d) illustrates that the Log-LFR distribution closely follows the observed data and outperforms alternative models such as the Exponential, Linear Failure Rate, and Rayleigh distributions. These visual findings are consistent with the numerical results, where the Log-LFR model achieves the lowest values for AIC, BIC, HQIC and the Kolmogorov–Smirnov statistic, affirming its superiority in modeling the given failure data.

### Dataset-II (aircraft windshields service time data)

Dataset-II comprises 63 service time observations of ranging from 0.046 to 5.140 time units, serving as a secondary validation dataset for the Log-LFR distribution model. This dataset provides an important comparative framework to assess the robustness and generalizability of the proposed distribution across different reliability scenarios. The analysis involved fitting four competing distributions Log-LFR, TLFR, LFR, Rayleigh (R), and Exponential (E) using maximum likelihood estimation methods to determine their relative performance in modelling the service patterns. The data points reflect the duration of windshield servicing operations, as documented by Murthy et al.^19^ in their Weibull analysis study. The comprehensive servicing duration measurements are displayed in the referenced Table 6.

**Table 6:** Service time of aircraft windshields data set.

0.046	1.436	2.592	0.140	1.492	2.600	0.150	1.580	2.670	0.248	1.719	2.717
0.280	1.794	2.819	0.313	1.915	2.820	0.389	1.920	2.878	0.487	1.963	2.950
0.622	1.978	3.003	0.900	2.053	3.102	0.952	2.065	3.304	0.996	2.117	3.483
1.003	2.137	3.500	1.010	2.141	3.622	1.085	2.163	3.665	1.092	2.183	3.695
1.152	2.240	4.015	1.183	2.341	4.628	1.244	2.435	4.806	1.249	2.464	4.881
1.262	2.543	5.140

**Table 7:** MLEs and goodness of fit values for aircraft windshields service time data.

Distribution	MLEs	AIC	BIC	AICc	HQIC	-lnL	K-S
Log-LFR	$\hat{\alpha } =0.2782$	174.1061	180.5355	174.5129	176.6348	84.0530	0.6109
	$$\hat{\beta } =0.2653$$						
	$$\hat{p} =3.5$$						
TLFR	$\hat{\beta }=0.2275$	202.8946	209.324	203.3014	205.4234	98.4473	0.0801
	$$\hat{\theta } =0.2435$$						
	$$\hat{\lambda } =-0.3849$$						
LFR	$\hat{\alpha } =0.1316$	201.5142	205.8005	201.7142	203.2000	98.7571	0.0931
	$$\hat{\beta } = 0.2469$$						
Rayleigh	$\hat{\alpha } =$0.75	186.5398	188.6829	186.6054	187.3827	92.2699	0.3345
Exponential	$\hat{\lambda }=0.4795$	222.5972	226.8835	222.7972	224.2830	109.2986	0.2077

The empirical results demonstrate that the Log-LFR distribution exhibits superior performance across multiple goodness-of-fit criteria. With parameter estimates of $\hat{\alpha } =0.2782$, $\hat{\beta } =0.2653$ and $\hat{p} =3.5$, the Log-LFR distribution model achieves the most favorable values for key information criteria which are mentioned Table 7, recording an AIC of 174.11, BIC of 180.54, AICc of 174.51, and HQIC of 176.63. The Log-LFR distribution model attains the highest log-likelihood value of −84.05, indicating superior data fitting capability compared to competing distribution models.

**Fig. 9:**
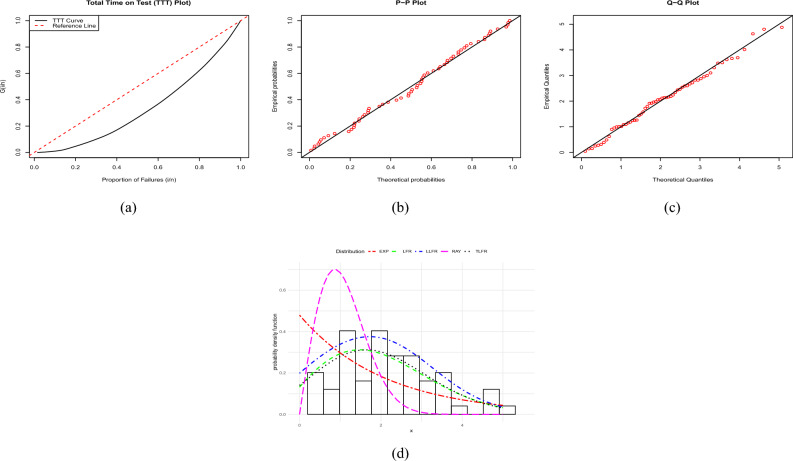
(**a**) TTT plot, (**b**) P-P plot, (**c**) Q-Q plot and (**d**) The fitted pdfs and histogram plot.

Figure 9 provides comprehensive graphical validation of the Log-LFR distribution’s performance on Dataset-II through four distinct diagnostic plots. The Total Time on Test (TTT) plot in Fig. 9(a) reveals the underlying service rate characteristics of the dataset, where the curve’s shape relative to the reference diagonal line indicates the nature of the hazard function a convex pattern suggests decreasing service rates over time. The P-P plot in Fig. 9(b) compares the empirical cumulative probabilities against the theoretical probabilities predicted by the Log-LFR model, with points closely following the diagonal line demonstrating excellent agreement between observed and expected values. The Q-Q plot in Fig. 9(c) similarly assesses model adequacy by plotting empirical quantiles against theoretical quantiles, where the linear relationship along the diagonal confirms that the Log-LFR distribution accurately captures the data’s distributional characteristics. Finally, Fig. 9(d) presents a comparative visualization of the fitted probability density functions overlaid on the data histogram, where the Log-LFR curve demonstrates superior alignment with the observed data pattern compared to the Exponential (E), LFR and Rayleigh (R) distributions. Collectively, these plots provide strong visual evidence supporting the statistical superiority indicated by the goodness-of-fit measures, confirming that the Log-LFR distribution effectively models the complex service patterns present in Dataset-II.

## Conclusion and future direction

The Log-LFR distribution is introduced as a generalization of the LFR distribution model. There are numerous statistical properties of this distribution such as the hazard rate, reversed hazard rate, mean residual life function, moments, moment-generating function, quantile function, order statistics and Rènyi entropy are investigated. Furthermore, the study investigated stochastic comparisons based on the usual stochastic ordering, with a particular emphasis on series systems that have the Log-LFR distributed components. The stochastic ordering results are validated by illustrative examples and counterexamples. The proposed model’s practical relevance is demonstrated through applications to two real datasets: aircraft windshield failure times (Dataset-I) and aircraft windshield service times (Dataset-II). In both cases, the Log-LFR distribution provides better goodness-of-fit, with lower AIC and BIC values compared to competing distribution models. Parameters are estimated using the maximum likelihood estimation method, and simulation studies suggest that the estimators are exhibit desirable asymptotic properties.

The present study has certain limitations. The parameter estimation has been carried out using the Maximum Likelihood Estimation method. Other estimation approach such as Bayesian estimation is not examined and can be considered in future research. The stochastic ordering results are developed under the assumption of independent random variables and focus mainly on magnitude-based orderings. Extending the analysis to dependent structures and variability-based orderings will provide deeper insights. Future studies addressing these aspects can enhance the model’s application in reliability and survival analysis.

## Data Availability

The datasets used in this study are provided in the manuscript, and also publicly available. Each dataset is cited with its corresponding source within the manuscript.

## Code availability

The R code used in this study are available at https://github.com/revathi04-mani/Log-linear-failure-rate-distribution-R-code.
